# Improving risk estimates for metabolically healthy obesity and mortality using a refined healthy reference group

**DOI:** 10.1530/EJE-17-0217

**Published:** 2017-05-30

**Authors:** Mark Hamer, William Johnson, Joshua A Bell

**Affiliations:** 1 School of SportExercise & Health Sciences, Loughborough University, Loughborough, UK; 2 MRC Integrative Epidemiology Unit at the University of BristolBristol, UK

## Abstract

**Objective:**

We aimed to re-examine mortality risk estimates for metabolically healthy obesity by using a ‘stable’ healthy non-obese referent group.

**Design:**

Prospective cohort study.

**Methods:**

Participants were 5427 men and women (aged 65.9 ± 9.4 years, 45.9% men) from the English Longitudinal Study of Ageing. Obesity was defined as body mass index ≥30 kg/m^2^ (vs non-obese as below this threshold). Based on blood pressure, HDL cholesterol, triglycerides, glycated hemoglobin and C-reactive protein, participants were classified as ‘healthy’ (0 or 1 metabolic abnormality) or ‘unhealthy’ (≥2 metabolic abnormalities).

**Results:**

Totally, 671 deaths were observed over an average follow-up of 8 years. When defining the referent group based on 1 clinical assessment, the unhealthy non-obese (hazard ratio (HR) = 1.22; 95% CI: 1.01, 1.45) and unhealthy obese (HR = 1.29; CI: 1.05, 1.60) were at greater risk of all-cause mortality compared to the healthy non-obese, yet no excess risk was seen in the healthy obese (HR = 1.14; CI: 0.83, 1.52). When we re-defined the referent group based on 2 clinical assessments, effect estimates were accentuated and healthy obesity was at increased risk of mortality (HR = 2.67; CI: 1.64, 4.34).

**Conclusion:**

An unstable healthy referent group may make ‘healthy obesity’ appear less harmful by obscuring the benefits of remaining never obese without metabolic dysfunction.

## Introduction

Termed ‘metabolically healthy obesity’, population-based studies have identified an obesity phenotype that is not accompanied by a clustering of adiposity-associated cardio-metabolic risk factors (1). Although tendencies for metabolic decline and for developing type 2 diabetes are becoming clear (1), associations of healthy obesity with outcomes related to cardiovascular disease and mortality are less consistent (2, 3). One explanation for these inconsistencies may be differences in the duration of follow-up used across studies, with those using shorter follow-up times tending to find no association between healthy obesity and outcomes (4, 5). Some studies have considered instability of healthy obesity itself as another potential explanation (6, 7, 8); yet little-to-no attention has focused on the importance of the group to which healthy obesity is being compared, usually defined as some form of ‘healthy non-obese’.

Studies that examine the risks of healthy obesity tend to rely on one-off measures of body mass index (BMI) and biomarkers of metabolic health. As a consequence, estimates of excess disease risk among healthy obese vs healthy non-obese adults may be obscured in at least two ways; one, by failing to distinguish those obese individuals who have been obese for many years from those who have only recently gained enough weight to become obese; and two, by failing to distinguish referent non-obese individuals who have had a BMI below the obese range for many years from those who only recently became obese or who used to be obese but had lost weight. These same obscurities would apply to markers of metabolic health, although these are known to track closely with changes in BMI, even within the strictly normal-weight range (9).

This study aimed to improve risk estimates for mortality among healthy obese adults by examining associations using a stable healthy non-obese referent group defined from repeat biomedical assessments.

## Subjects and methods

### Study sample and procedures

The English Longitudinal Study of Ageing (ELSA) is an ongoing cohort study that contains a nationally representative sample of free-living men and women born on or before February 29, 1952 (10). Data collected in 2004–2005 were used as the baseline for the present analysis as this was the first occasion; clinical data were measured objectively by a nurse. An identical clinical assessment was repeated 4 years later (2008–2009). Individual participant data were linked with death records from National Health Service registries for all consenting respondents (96.5% of the sample) up to February 2012. Participants gave full informed written consent to participate in the study, and ethical approval was obtained from the London Multi-Centre Research Ethics Committee.

### Clinical measurements

Nurses collected anthropometric data (weight, height), blood pressure (BP) and non-fasting blood samples using standard protocols. Body weight was measured without shoes and in light clothing using Tanita electronic scales, and height was measured using a stadiometer with the Frankfort plane in the horizontal position. BMI was calculated as weight (kilograms)/height (meters) squared. Systolic and diastolic BP was measured with an Omron HEM-907 blood pressure monitor three times in the sitting position after 5-min rest between each reading. The initial reading was discarded, and an average of the second and third BP recordings was used for the present analyses. Blood samples were analyzed for high-sensitivity C-reactive protein (CRP), high-density lipoprotein (HDL) cholesterol, triglycerides and glycated hemoglobin (HbA1c). Detailed information on the technicalities of the blood analysis, the internal quality control and the external quality assessment for the laboratory have been described elsewhere (11). Additional data were collected on physician diagnosed conditions (hypertension, diabetes) and medication use.

### Covariates at baseline

Age and sex were recorded in 2004–2005. Ethnicity was not considered as the sample was ethnically homogeneous (93.9% white-British). Health behaviors included cigarette smoking (current, previous or non-smoker), the frequency of participation in light, moderate and vigorous physical activities (more than once per week, once per week, one to three times per month, hardly ever), any reported chronic illness and depressive symptoms (a score of 3 or higher out of 8 on the 8-item Centre of Epidemiological Studies Depression scale (12)). Wealth was used as a measure of socioeconomic status, as this has been shown to best capture the material resources available to older adults (13). Wealth was calculated as net of debt and included the total value of the participant’s home (excluding mortgage), financial assets such as savings, business assets and physical wealth such as artwork or jewelry.

### Statistical analyses

We used conventional criteria to define obesity (BMI ≥30 kg/m^2^). Underweight participants (BMI <18.5 kg/m^2^) were excluded to prevent possible reverse causation (as underweight is often a marker of serious illness in older adults). A healthy metabolic status was based on independently proposed criteria (14), and according to availability of data, defined as having less than 2 of the following 5 metabolic risk factors: high BP (BP ≥130/85 mmHg, or hypertension diagnosis or use of anti-hypertensive medication), impaired glycemic control (HbA1c >6.0% (42.1 mmol/mol) or doctor’s diagnosed diabetes), systemic inflammation (CRP ≥ 3 mg/L), low HDL cholesterol (<1.03 mmol/L in men and <1.30 mmol/L in women) and high triacylglycerol (≥1.7 mmol/L). Participants were then categorized into 1 of 4 groups: ‘healthy non-obese’, ‘unhealthy non-obese’, ‘healthy obese’ and ‘unhealthy obese’. We used Cox proportional hazards regression models to examine associations between metabolic-BMI group and mortality, with healthy non-obese based on the 2004–2005 assessment. Age at death was recorded, and years were the time scale for the follow-up. For consenting participants with no record of an event, the data were censored at February 2012. The proportional hazards assumption was examined by using plots of the Nelson-Aalen cumulative hazard estimates. We estimated models that were initially adjusted for age and sex. The final models were additionally adjusted for physical activity, smoking, depressive symptoms (CES-D >3) and chronic illness, wealth. These covariates were selected a priori based on previous literature (4, 6, 8). These analyses were repeated with the healthy non-obese referent group refined to include only participants who met criteria for a healthy status and were non-obese in both 2004–2005 and 2008–2009. All other participants remained assigned to their baseline categories, and those from the original referent category that transitioned into unhealthy/obese groups (*n* = 515) or did not provide data at follow-up (*n* = 994) were removed. Deaths in the first 4 years of follow-up were removed in order to retain consistency with the new referent category. All models were repeated in sensitivity analyses using a more stringent definition of metabolic health, defined as having 0 of 5 risk factors. A two-sided *P* value of 0.05 was used to indicate statistical significance. All analyses were conducted using SPSS version 22 (SPSS).

## Results

A total of 8688 participants (82% of participants at the first point of contact in 2002–2003) attended the clinical assessment in 2004–2005. The present study reports only on those that consented and were eligible and able to give blood (*n* = 5903); this excluded men and women with clotting and bleeding disorders, or taking anti-coagulant medication. After excluding 79 participants that did not provide consent to linkage of mortality records, and a further 397 because of missing data, the final analytic sample comprised 5427 men and women (aged 65.9 ± 9.4 years, 45.9% men).

Descriptive characteristics of the sample are displayed in Table 1. Around 46.0% of the sample were characterized as ‘healthy non-obese’, 25.2% as ‘unhealthy non-obese’, 9.5% as ‘healthy obese’ and 19.3% as ‘unhealthy obese’. The healthy non-obese were generally wealthier than other participants and displayed better health behavior including higher physical activity levels, fewer depressive symptoms and chronic illnesses.

**Table 1:** Baseline characteristics of the sample (*n* = 5427).

	**Metabolically healthy non-obese** (*n* = 2503)	**Metabolically unhealthy non-obese** (*n* = 1364)	**Metabolically healthy obese** (*n* = 514)	**Metabolically unhealthy obese** (*n* = 1046)
Age (years)	65.5 ± 9.5	67.7 ± 9.6	64.3 ± 8.8	65.6 ± 9.0
Men (%)	44.6	52.5	43.8	41.4
Depressive symptoms (% CES-D >3)	11.6	14.3	13.2	16.6
Longstanding illness (%)	44.2	58.3	46.9	65.0
Current smokers (%)	14.1	20.7	9.9	16.3
Vigorous physical activity (% at least once/week)	37.0	26.4	28.4	20.7
Lowest wealth quintile (%)	10.6	15.2	13.4	21.6
Body mass index (kg/m^2^)	25.1 ± 2.7	26.4 ± 2.4	32.8 ± 2.9	34.1 ± 3.9
HDL cholesterol (mmol/L)	1.67 ± 0.37	1.37 ± 0.36	1.54 ± 0.30	1.34 ± 0.32
Triglycerides (mmol/L)	1.37 ± 0.71	2.28 ± 1.06	1.54 ± 0.73	2.42 ± 1.74
HbA1c (%)	5.39 ± 0.38	5.77 ± 0.86	5.47 ± 0.41	5.95 ± 0.95
C-reactive protein (mg/L)	1.93 ± 3.41	5.11 ± 7.73	2.90 ± 4.48	6.26 ± 7.59
Systolic BP (mmHg)	130.5 ± 17.1	139.5 ± 18.7	135.8 ± 15.8	140.5 ± 18.3
Diastolic BP (mmHg)	73.2 ± 9.8	76.2 ± 11.9	75.9 ± 8.6	79.1 ± 11.6

A total of 671 deaths were observed over an average follow-up of 8 years. In covariate-adjusted models that defined the healthy non-obese referent group based on 2004–2005 only (Table 2), the unhealthy non-obese (hazard ratio (HR) = 1.22; 95% CI: 1.01, 1.45) and unhealthy obese (HR = 1.29; CI: 1.05, 1.60) were at greater risk of mortality, although no excess risk was observed in the healthy obese (HR = 1.14; CI: 0.83, 1.52). A similar pattern emerged for death from cardiovascular diseases (CVD) although significant excess risk remained only among the unhealthy obese in the final model. Medication (including anti-hypertensive, lipid lowering and diabetes medications) was used in 35.9% of the cohort at baseline. The results were not substantially different after controlling for medication use (Supplementary Table 1, see section on supplementary data given at the end of this article).

**Table 2:** Cox proportional hazards regression for associations of obesity, metabolic health and mortality, with referent healthy non-obese group defined by status in 2004–2005 only (*n* = 5427).

		HR (95% CI)	
**Baseline metabolic health/obesity status**	**Number of deaths/total *n***	Model 1	Model 2
All deaths			
Healthy non-obese	262/2503	1.00 (ref)	1.00 (ref)
Unhealthy non-obese	215/1364	1.35 (1.12, 1.61)	1.22 (1.01, 1.45)
Healthy obese	52/514	1.20 (0.89, 1.62)	1.14 (0.83, 1.52)
Unhealthy obese	142/1046	1.51 (1.23, 1.86)	1.29 (1.05, 1.60)
CVD deaths			
Healthy non-obese	62/2503	1.00 (ref)	1.00 (ref)
Unhealthy non-obese	54/1364	1.43 (1.00, 2.06)	1.29 (0.89, 1.87)
Healthy obese	9/514	0.98 (0.49, 1.97)	0.92 (0.45, 1.86)
Unhealthy obese	38/1046	1.91 (1.27, 2.88)	1.57 (1.03, 2.40)

Model 1 adjusted for age and sex. Model 2 adjusted for age, sex, wealth, physical activity, smoking, depressive symptoms, chronic illness.

During 4 years of follow-up, 65.8% of healthy non-obese participants remained stable in this category (Supplementary Table 2). In covariate-adjusted models that re-defined the healthy non-obese referent group based on data from both 2004–2005 and 2008–2009, effect estimates for mortality were accentuated in the unhealthy non-obese and obese groups, and the healthy obese were also at increased risk (HR = 2.67; CI: 1.64, 4.34) (Table 3).

**Table 3:** Cox proportional hazards regression for associations of obesity, metabolic health and mortality, with referent healthy non-obese group based on status in both 2004–2005 and 2008–2009 (*n* = 3868).

		HR (95% CI)	
**Baseline metabolic health/obesity status**	**Number of deaths/total *n***	Model 1	Model 2
All deaths			
Stable healthy non-obese^†^	27/992	1.00 (ref)	1.00 (ref)
Unhealthy non-obese	186/1335	3.52 (2.34, 5.28)	2.99 (1.98, 4.52)
Healthy obese	43/505	2.98 (1.84, 4.83)	2.67 (1.64, 4.34)
Unhealthy obese	132/1036	4.20 (2.78, 6.36)	3.50 (2.28, 5.35)
CVD deaths			
Stable healthy non-obese^†^	5/992	1.00 (ref)	1.00 (ref)
Unhealthy non-obese	47/1335	4.15 (1.64, 10.49)	3.64 (1.42, 9.33)
Healthy obese	5/505	1.79 (0.52, 6.19)	1.55 (0.44, 5.42)
Unhealthy obese	35/1036	5.78 (2.26, 14.78)	4.67 (1.78, 12.22)

Model 1 adjusted for age and sex. Model 2 adjusted for age, sex, wealth, physical activity, smoking, depressive symptoms and chronic illness.

^†^Reference group contains participants that remained metabolically healthy non-obese over four-year follow-up. Participants from referent category that transitioned into unhealthy groups (*n* = 515) or did not provide data at follow-up (*n* = 994) were removed. Deaths from first 4 years of follow-up were removed in order to retain consistency with referent category.

In sensitivity analyses, we used the more stringent definition of metabolic health (based on 0 of 5 risk factors) although results remained largely unchanged (Supplementary Table 3). Our original criterion for metabolic health was based on the clinically valid CRP threshold of 3 mg/L (15). We also re-defined metabolic health using the 90th percentile for CRP (8.2 mg/L in our sample), as used by others (14), although the results were not substantially changed (Supplementary Table 4).

## Discussion

The aim of this study was to examine associations between metabolically healthy obesity phenotype and mortality. Unlike previous studies we defined the healthy non-obese referent category from repeated biomedical assessments in order to ensure that risk among healthy obese adults was compared with that of adults who remained never obese without metabolic risk factors over an extended period of time. Using this refined referent category, our results suggest that excess risk of mortality among unhealthy non-obese and unhealthy obese groups is greater than previously thought, and that risk is indeed elevated among the healthy obese.

Previous studies (2, 3, 4, 5) had defined the referent category (‘healthy non-obese’) based on clinical assessments made at a single point in time, thus potentially making ‘healthy obesity’ appear less harmful by making comparisons with a group that is heterogeneous with respect to obesity exposure. Indeed, an unstable referent category may create bias in several ways, such as through containing individuals who were recently obese but had lost weight due to the onset of disease. This issue is still possible in the groups as defined in our study, for example, if adults with very high BMI lost weight in response to disease yet still fell within the obese range of BMI. The number of individuals with very high BMI, however, was small in our sample (7.5% of obese above BMI 40 kg/m^2^); most were just above the obesity threshold of 30 kg/m^2^ (72.5% of the obese being between 30 and 35 kg/m^2^). Nevertheless, the older age of participants in the present study remains a limitation, given that associations between BMI and mortality risk are more prone to reverse causation by way of subclinical disease-induced weight loss than are such associations when examined among younger adults (16).

The few studies that have made use of multiple observations of BMI over the life course to study disease outcomes suggest that each additional observation enhances predictive value, with future morbidity being a positive function of the duration of obesity (17, 18, 19, 20, 21). This has been recently applied within the ‘healthy obesity’ paradigm to demonstrate that greater duration of obesity was associated with higher likelihood of developing the metabolic syndrome (22).

This study has its limitations. We were only able to utilize biomedical data from two visits as weight histories prior to baseline were not available. Given a lack of consensus for the definition of metabolically healthy obesity, we defined metabolic health using an adaptation of previous criteria (14) according to availability of data. The decision to use a categorization of 0 or 1 metabolic risk factor to define a metabolically healthy status was to allow for consistency with previous work [14] and for clinical relevance. Sensitivity analyses using a more stringent definition based on zero metabolic risk factors to define metabolic health produced similar results, yet were necessarily based on smaller numbers of participants given that most ‘healthy’ obese adults present with 1 risk factor.

In conclusion, using repeat biomedical assessments in order to define a referent group that remained never obese without metabolic risk factors for an extended period of time, our results suggest that the risk of mortality is far greater among unhealthy non-obese and unhealthy obese adults than previously thought, and is indeed elevated among the healthy obese. Previous studies may have underestimated the detrimental effects of ‘healthy obesity’ by obscuring the benefits of remaining never obese without metabolic risk factors.

## Supplementary Material

Supporting Table 1

Supporting Table 2

Supporting Table 3

Supporting Table 4

## Declaration of interest

The authors declare that there is no conflict of interest that could be perceived as prejudicing the impartiality of this study.

## Funding

The data were made available through the UK Data Archive. M Hamer acknowledges support from the National Institute for Health Research (NIHR) Leicester Biomedical Research Centre, which is a partnership between University Hospitals of Leicester NHS Trust, Loughborough University and the University of Leicester. J A Bell is supported by CRUK (C18281/A19169). W Johnson is supported by an Medical Research Council (MRC) New Investigator Research Grant (MR/P023347/1). The funders had no role in the study design; in the collection, analysis and interpretation of data; in writing of the report; or in the decision to submit the paper for publication. The developers and funders of ELSA and the Archive do not bear any responsibility for the analyses or interpretations presented here.

## Author contribution statement

M Hamer had full access to the data and takes responsibility for the integrity and accuracy of the results. M Hamer drafted the paper, performed analyses and designed the study. W Johnson and J A Bell contributed to the concept and design of the study and critical revision of the manuscript.

## Data sharing statement

Full ELSA data are available at the UK data archive http://www.data-archive.ac.uk/.
