# Supplementary material for: Improving risk estimates for metabolically healthy obesity and mortality using a refined healthy reference group
Source: Eur J Endocrinol. 2017 May 30;177(2):169–74. doi: 10.1530/EJE-17-0217 (PMC5967883; doi:10.1530/EJE-17-0217)
Supplement: Supporting Table 1 [file eje-177-169-t001.pdf]

Supplementary analyses

Table S1. Cox proportional hazards regression for associations of obesity, metabolic health and mortality, with referent healthy non-obese group defined by status in 2004-05 only (n=5,427). Models additionally adjusted for medication.

Baseline metabolic health/obesity status	All Deaths / N	Model 1 HR (95% CI)	Model 2 HR (95% CI)
Healthy non-obese	262/2503	1.00 (ref)	1.00 (ref)
Unhealthy non-obese	215/1364	1.35 (1.12, 1.61)	1.31 (1.08, 1.58)
Healthy obese	52/514	1.20 (0.89, 1.62)	1.17 (0.86, 1.58)
Unhealthy obese	142/1046	1.51 (1.23, 1.86)	1.38 (1.11, 1.71)
	**CVD deaths/N**		
Healthy non-obese	62/2503	1.00 (ref)	1.00 (ref)
Unhealthy non-obese	54/1364	1.43 (1.00, 2.06)	1.40 (0.95, 2.06)
Healthy obese	9/514	0.98 (0.49, 1.97)	0.95 (0.47, 1.93)
Unhealthy obese	38/1046	1.91 (1.27, 2.88)	1.68 (1.09, 2.59)

Model 1 adjusted for age and sex

Model 2 adjusted for age, sex, wealth, physical activity, smoking, depressive symptoms, chronic illness, medication (lipid lowering, anti-hypertensive, diabetes medication).
