# Supplementary material for: Improving risk estimates for metabolically healthy obesity and mortality using a refined healthy reference group
Source: Eur J Endocrinol. 2017 May 30;177(2):169–74. doi: 10.1530/EJE-17-0217 (PMC5967883; doi:10.1530/EJE-17-0217)
Supplement: Supporting Table 2 [file eje-177-169-t002.pdf]

Supplementary analyses

Table S2. Change in metabolic health/obesity status over 4 years

Baseline		4- year follow-up				Total
		MHNO	MUNO	MHO	MUO	
	MHNO	994	406	49	60	1509
	MUNO	185	499	7	73	764
	MHO	25	19	107	129	280
	MUO	16	46	58	417	537
Total		1220	970	221	679	3090

Healthy non-obese (MHNO) ; unhealthy non-obese (MUNO); Healthy obese (MHO); Unhealthy obese (MUO)
