# Supplementary material for: Improving risk estimates for metabolically healthy obesity and mortality using a refined healthy reference group
Source: Eur J Endocrinol. 2017 May 30;177(2):169–74. doi: 10.1530/EJE-17-0217 (PMC5967883; doi:10.1530/EJE-17-0217)
Supplement: Supporting Table 3 [file eje-177-169-t003.pdf]

Supplementary analyses

Table S3. Cox proportional hazards regression for associations of obesity, metabolic health and mortality: sensitivity analyses using zero risk factors to define 'metabolically healthy' and referent healthy non-obese group based on 2004-05 only (n=5,427).

**Baseline metabolic health/obesity status**	**All Deaths / N**	**Model 1 HR (95% CI)**	**Model 2 HR (95% CI)**
Healthy non-obese	90/1155	1.00 (ref)	1.00 (ref)
Unhealthy non-obese	387/2712	1.61 (1.28, 2.02)	1.49 (1.18, 1.87)
Healthy obese	14/142	1.46 (0.83, 2.57)	1.39 (0.79, 2.44)
Unhealthy obese	156/1348	1.73 (1.33, 2.25)	1.49 (1.14, 1.95)
	**CVD deaths/N**		
Healthy non-obese	15/1155	1.00 (ref)	1.00 (ref)
Unhealthy non-obese	101/2712	2.60 (1.51, 4.47)	2.42 (1.40, 4.19)
Healthy obese	4/142	2.70 (0.89, 8.12)	2.63 (0.87, 7.98)
Unhealthy obese	37/1348	2.90 (1.58, 5.34)	2.46 (1.33, 4.56)

Model 1 adjusted for age and sex

Model 2 adjusted for age, sex, wealth, physical activity, smoking, depressive symptoms, chronic illness.
