# Supplementary material for: Improving risk estimates for metabolically healthy obesity and mortality using a refined healthy reference group
Source: Eur J Endocrinol. 2017 May 30;177(2):169–74. doi: 10.1530/EJE-17-0217 (PMC5967883; doi:10.1530/EJE-17-0217)
Supplement: Supporting Table 4 [file eje-177-169-t004.pdf]

Supplementary analyses

Table S4. Cox proportional hazards regression for associations of obesity, metabolic health and mortality, with referent healthy non-obese group defined by status in 2004-05 only (n=5,427). Categories re-defined using modified criteria (90th percentile) for C-reactive protein.

**Baseline metabolic health/obesity status**	**All Deaths / N**	**Model 1 HR (95% CI)**	**Model 2 HR (95% CI)**
Healthy non-obese	300/2876	1.00 (ref)	1.00 (ref)
Unhealthy non-obese	177/1085	1.35 (1.16, 1.64)	1.31 (1.07, 1.59)
Healthy obese	71/728	1.18 (0.91, 1.53)	1.13 (0.87, 1.47)
Unhealthy obese	99/794	1.44 (1.15, 1.81)	1.31 (1.03, 1.67)
	**CVD deaths/N**		
Healthy non-obese	73/2876	1.00 (ref)	1.00 (ref)
Unhealthy non-obese	43/1085	1.33 (0.91, 1.94)	1.40 (0.95, 2.06)
Healthy obese	14/728	1.08 (0.61, 1.92)	0.95 (0.47, 1.93)
Unhealthy obese	27/794	1.84 (1.18, 2.88)	1.68 (1.09, 2.59)

Model 1 adjusted for age and sex

Model 2 adjusted for age, sex, wealth, physical activity, smoking, depressive symptoms, chronic illness, medication (lipid lowering, anti-hypertensive, diabetes medication).
